# Medication Dispensing Patterns Following Teleconsultations in France From January 2021 to June 2023: A Retrospective Cohort Study Using the National Health Data System

**DOI:** 10.34172/ijhpm.9215

**Published:** 2026-05-24

**Authors:** Julie Rigoureau, François Bettega, Marc Chanelière, Teddy Novais, Stéphane Sanchez, Stéphanie Sidorkiewicz, Aurélie Moskal, Marie Viprey

**Affiliations:** ^1^Research on Healthcare Performance (RESHAPE), INSERM U1290, Université Claude Bernard Lyon 1, Lyon, France.; ^2^Collège universitaire de Médecine Générale, Université Lyon 1, Villeurbanne, France.; ^3^Service Pharmacie, Hôpital des Charpennes, Hospices Civils de Lyon, Villeurbanne, France.; ^4^Pôle Territorial Santé Publique, Hôpitaux Champagne Sud, Troyes, France.; ^5^Faculté de Médecine, Université de Reims Champagne Ardennes, Reims, France.; ^6^Centre for Research in Epidemiology and Statistics (CRESS), Université Paris Cite and Université Sorbonne Paris Nord, Inserm, INRAE, Paris, France.; ^7^Department of General Practice, Université Paris Cité, Paris, France.; ^8^Hospices Civils de Lyon, Service des Données de Santé, Lyon, France.

**Keywords:** Remote Consultation, Teleconsultation, Drug Utilization, Medication, Pharmacoepidemiology

## Abstract

**Background:**

Teleconsultation services grew significantly in France during the COVID-19 pandemic. Reimbursed teleconsultations increased by a hundredfold between 2019 and 2021, establishing it as an emerging healthcare delivery modality. However, medication dispensing patterns following teleconsultations remain largely unexplored. This study aimed to characterize post-teleconsultation medication dispensing patterns between 2021 and 2023.

**Methods:**

A retrospective cohort study was conducted using data from the French health database. The study included all teleconsultations conducted that resulted in at least one medication dispensing event (TLCs) between January 1, 2021, and June 30, 2023. We described patient demographics, prescriber characteristics, and dispensed medication.

**Results:**

During the study period, 11 202 364 TLCs (34.3%) out of 33 853 911 resulted in medication dispensing. Among these 11 202 364 TLCs, the patient population predominantly comprised females (64.4%), individuals without long-term medical conditions (86.4%), with a mean age of 42.2 years (SD ± 20.8). Teleconsultations were mainly conducted by liberals’ physicians (86.8%) and health centre-employed practitioners (12.1%). The use of teleconsultation platforms increased fivefold between the first semesters 2021 (4.2%) and 2023 (28.2%). Teleconsultations resulted in medications targeting the nervous system (53.1%), digestive system (28.3%), and respiratory system (21.8%). Two contexts of dispensing emerged: dispensing with chronic indication (42.8% of TLCs) characterized by a long median time and several dispensing, and dispensing with an acute indication (55.6%), characterized by single dispensing with a 0-1 day between teleconsultation and dispensing.

**Conclusion:**

One third of the teleconsultations resulted in medication dispensing, mainly for young, urban patients with good access to care, in contrast to the objective as described in the French Law. Dispensing patterns revealed two distinct modalities, acute symptomatic and chronic disease management. Certain classes of drugs dispensed (antibiotics, opioids, benzodiazepines) may raise questions about appropriate dispensing practices in teleconsultations.

## Introduction

**Key Messages**

**Implications for policy makers**

- Most teleconsultations were attended by patients with good care access and a preferred doctor, diverging from the intended French Law goals of addressing underserved areas.
- Some dispensing involved treatments with misappropriation potential (opioids) or requiring diagnostic testing (antiinfectives), raising concerns about best dispensing practices.
- Private teleconsultation companies, which increased by fivefold during the study period, need to be studied in greater detail in terms of compliance with health authority guidelines.

**Implications for the public**

Between 2021 and 2023, teleconsultations became an established mode of healthcare access for the French population. They were used in 55.6% of cases for acute illnesses, often resulting in the prescription of treatments like antibiotics, which ideally require at least a diagnostic test. Teleconsultations were also utilized in the management of chronic conditions. Teleconsultation appears to meet a rapid healthcare access need, which may explain why the majority of teleconsultations were attended in urban areas with good healthcare access. Certain teleconsultation utilisation, such as those involving private companies or treatment initiation, warrant further investigation to ensure patient safety and quality of care.

Telemedicine was defined by the World Health Organization (WHO) in 1997 as “the practice of medical interventions facilitated through interactive data communication technologies.”^1^ It has developed worldwide at varying rates, since 1970s.^2,3^ In France, telemedicine was legally established in 2010, in order to improve healthcare accessibility in areas with low healthcare supply.^4^ Teleconsultation was defined as a procedure designed to enable a medical professional to give a remote consultation to a patient.^4^ The reimbursement of teleconsultations to the entire population by health insurance was ratified in September 2018.^5^ At first, teleconsultations remained marginal in France, in contrast to other countries with a greater acculturation to teleconsultation practices.^6,7^ The COVID-19 crisis contributed to its expansion in France, with 13.5 million teleconsultations reimbursed in 2020^8^ ensuring a relative continuity of care during the COVID-19’s restrictions.^9,10^

 Over the COVID-19 period, teleconsultations and the associated prescriptions have become a significant aspect of the healthcare system, sometimes becoming the default prescription form during the various lockdowns.^11,12^ Studies on treatments dispensed after teleconsultation focused on specific classes of drugs, which makes it difficult to have an overview of the teleconsultation’s impact on dispensing.^13-15^ Since 2021, teleconsultations has become an integral part of professional’s and patient’s practices.^16^

 Although this mode of care could be beneficial for improving the follow-up of chronic illnesses, or for treating acute conditions,^6,17,18^ some situations are not suited for teleconsultation, such as those requiring auscultation, biological tests or treatment initiation.^19^ This potentially inappropriate practices may lead to patient safety and treatment efficiency issues. Better characterizing the context of dispensing and the profiles of patients and prescribers could help adapt the therapeutic strategy and optimize the use of this healthcare resource. To our knowledge, no such study has yet been conducted in France. The aim of the present study was therefore to provide an overview of dispensing situations following teleconsultation in France between January 1, 2021 and June 30, 2023, describing the characteristics of patients, prescribers, and dispensing.

## Methods

 The study was conducted in accordance with the reporting of studies conducted using observational routinely collected health data statement for pharmacoepidemiology (RECORD-PE)^20^ (Supplementary file 1).

###  Study Design, Population, and Follow-up

 A retrospective cohort study was conducted using data from the French nationwide health insurance databases (Système National des Données de Santé, *SNDS*). We included all the teleconsultations executed by physician between January 1, 2021 and June 30, 2023, resulting in at least one dispensing within 6 months of the teleconsultation. Teleconsultations attended by patients with inconsistent demographic data were excluded.

###  Data Source

 The* SNDS* covers 98% of the French population^21^ and contains individual pseudonymized socio-demographic data, all reimbursement data for primary care (physician or paramedical visits and procedures and drugs dispensed [without indications]) and inpatient care (diagnoses coded with International Classification of Diseases, 10th Revision (ICD-10), date and duration of hospitalization and procedures).

###  Outcomes

 Patient’s characteristics were described at the date of teleconsultation: age, sex, and healthcare accessibility. We used two indicators to assess it, first the number of teleconsultations per 1000 inhabitants was described by geographic area, calculated by dividing the number of teleconsultations by the population of each geographic area,^22^ and second general practitioners (GPs) healthcare accessibility at patient’s residence postcode, defined by the localized potential accessibility (LPA) index.^23,24^ This ecological index is calculated through the number, type, and complexity of acts performed by GP, weighted according to their workload and practice context. The socio-economic status of patients was described with two proxies, first the French Deprivation index (*FDep*), which is an ecological index of deprivation,^25^ then the access to complementary solidarity health (*C2S/ACS*), which constitutes a strong individual proxy of low income. Finally, the medical characteristics were described by the presence of a long-term disease (*LTD*) allowing full reimbursement of care by healthcare insurance for the specific condition, but not for all healthcare contacts.

 The prescribers were described according to their practice mode, medical specialties (not available for hospital-based practitioner), and their designation status as the patient’s preferred doctor. Practice mode of prescribers could be liberals, public or private hospital physicians and health centre-employed practitioners including teleconsultation platforms. Teleconsultation commercial platforms, whose provide teleconsultations carried out by doctors they employ,^26^ were identified using the FINESS number (company’s identification number).

 Treatment characteristics were reported by Anatomical Therapeutic Chemical Classification (ATC) class which organizes drugs into groups at five hierarchical levels.^27^ We presented treatments at the first level (ATC-1), corresponding to the 14 main anatomical or pharmacological groups, at the third level (ATC-3), representing chemical subgroups, and at the fifth level (ATC-5), corresponding to the individual chemical substance. We described the number of different ATC-5 dispensed by teleconsultation and the number of different dispensing dates. For each ATC-1 and ATC-3 classes, the following criteria have been described: number of teleconsultations with at least one dispensing of the classes and median time in days between teleconsultation date and first dispensing. In addition, number of dispensing of the same class following teleconsultation were calculated for ATC-3 classes. We have described the ATC-1 classes for that accounted for at least 0.1% of teleconsultations, and the top 10 ATC-3 classes most dispensed.

 Teleconsultations were classified as acute or chronic context based on the ATC-3 classification of dispensed medications. We established the classification according to the authorized therapeutic indications as specified in the Marketing Authorization documentation. A teleconsultation was designated as chronic if it resulted in the prescription of at least one medication categorized for chronic treatment (Supplementary file 2, Table S1).

###  Statistical Analysis

 We described categorical variables using numbers and percentages and quantitative variables using mean and standard deviation, median and quartiles. Patient’s and prescriber’s characteristics were described over the entire period and by semester. To assess the association between the number of teleconsultations standardized on the population^28^ and LPAs by department, we calculated a Pearson correlation coefficient. We have described the ATC-1 classes for that accounted for at least 0.1% of teleconsultations, and the top 10 ATC-3 classes most dispensed. Treatment characteristics were described in total, per age group (under 18s, 18-59s, 60s, and over) and per prescriber’s characteristics (practice mode and specialties). Analyses were performed using SAS^®^ software version 7.15 HF9 and R software version 4.1.2.

## Results

###  Teleconsultations’ Characteristics

 Between January 2021 and June 2023, 11 202 364 teleconsultations resulting in dispensed medications (TLC) were attended by 6 158 417 patients. Most patients (66.3%) attended only one TLC, while 5.9% attended five or more. After a decrease from the first semester of 2021 (N = 8 188 764) to the first semester of 2022 (N = 5 832 181), monthly teleconsultation volumes stabilized from May 2022 onwards, averaging one million consultations per month. Proportion of teleconsultations resulting in medication dispensing followed a similar trend, maintaining a ratio of 30%.

###  Patients’ Socio-demographic and Medical Characteristics 

 Among the TLC, 64.4% were attended to female patients and patients mean age was 42.2 years (±20.8). In the first semester of 2021, the over-60s attended 25.9% of teleconsultations, then this proportion declines, stabilizing at around 19% over the 2022 and 2023 half-years (Table 1).

**Table 1:** Socio-demographic and Medical Characteristics of Patients at the Time of the Teleconsultation

	**2021**		**2022**		**2023**	**Total**
	**S1**	**S2**	**S1**	**S2**	**S1**	
**Teleconsultations, N**^a^	2 648 520	1 917 246	2 517 230	2 061 691	2 057 678	11 202 364
Male Sex, No. (%)	955 103 (36.1)	679 885 (35.5)	895 666 (35.6)	658 805 (32.0)	730 768 (35.5)	3 991 150 (35.6)
Age						
Age, mean ± SD	45.5 ± 21.3	41.4 ± 20.6	41.9 ± 20.6	40.8 ± 20.6	40.5 ± 20.0	42.2 ± 20.8
Age group, No. (%)						
≤11 years	156 642 (5.9)	144 616 (7.5)	186 894 (7.4)	161 921 (7.9)	144 495 (7.0)	794 568 (7.1)
12–17 years	73 448 (2.8)	56 282 (2.9)	79 784 (3.2)	65 216 (3.2)	60 683 (2.9)	335 413 (3.0)
18–39 years	861 576 (32.5)	747 057 (39.0)	948 180 (37.7)	824 555 (40.0)	876 525 (42.6)	4 257 893 (38.0)
40–59 years	871 459 (32.9)	598 974 (31.2)	805 526 (32.0)	621 924 (30.2)	613 348 (29.8)	3 511 231 (31.3)
60–79 years	501 054 (18.9)	282 679 (14.7)	380 825 (15.1)	298 584 (14.5)	277 181 (13.5)	1 740 323 (15.5)
≥80 years	184 341 (7.0)	87 638 (4.6)	116 021 (4.6)	89 491 (4.3)	85 446 (4.2)	562 937 (5.0)
C2S/ACS, No. (%)	207 912 (10.4)	148 583 (9.5)	193 356 (9.4)	170 659 (10.1)	188 193 (11.0)	908 703 (10.1)
FDEp, No. (%)						
Q1, less deprived	724 413 (27.4)	568 748 (29.7)	730 812 (29.0)	588 501 (28.5)	588 091 (28.6)	3 200 565 (28.6)
Q2	561 966 (21.2)	412 727 (21.5)	543 644 (21.6)	433 765 (21.0)	431 182 (21.0)	2 383 284 (21.2)
Q3	488 845 (18.5)	337 916 (17.6)	450 793 (17.9)	362 541 (17.6)	352 803 (17.1)	1 992 898 (17.8)
Q4	409 271 (15.5)	275 570 (14.4)	375 253 (14.9)	316 092 (15.3)	317 167 (15.4)	1 693 353 (15.1)
Q5, most deprived	398 090 (15.0)	268 786 (14.0)	356 113 (14.1)	316 789 (15.4)	311 986 (15.2)	1 651 764 (14.7)
Missing data	65 935 (2.5)	53 499 (2.8)	60 615 (2.4)	44 003 (2.1)	56 449 (2.7)	28 0501 (2.5)
LTD, No. (%)	789 898 (30.2)	502 496 (26.2)	628 066 (25.0)	523 403 (25.4)	513 320 (25.0)	2 957 183 (26.4)
LPA index for GPs, No. (%)						
Decile 1 (lower)	61 176 (2.3)	49 086 (2.6)	66 570 (2.6)	62 814 (3.0)	66 076 (3.2)	305 722 (2.7)
Decile 9 or 10	1 015 638 (38.3)	698 132 (36.4)	916 838 (36.4)	692 568 (33.6)	656 427 (31.9)	3 979 603 (35.5)
Residential area per 1000 inhabitants, N (%)						
Large urban areas	50 (21.0)	38 (23.2)	49 (22.3)	39 (21.2)	39 (21.2)	216 (21.8)
Intermediate urban areas	37 (15.6)	26 (15.8)	35 (15.9)	29 (15.8)	29 (15.8)	155 (15.6)
City rings	33 (13.9)	22 (13.4)	30 (13.6)	26 (14.1)	26 (14.1)	162 (16.3)
Small cities	39 (16.4)	27 (16.5)	36 (16.4)	30 (16.3)	29 (15.8)	138 (13.9)
Rural towns	30 (12.6)	20 (12.2)	27 (12.3)	23 (12.5)	23 (12.5)	124 (12.5)
Rural, scattered settlements	28 (11.8)	18 (11)	25 (11.4)	21 (11.4)	21 (11.4)	113 (11.4)
Rural, widely scattered settlements	21 (8.8)	13 (7.9)	18 (8.2)	16 (8.7)	17 (9.2)	85 (8.6)

Abbreviations:SD, standard deviation;C2S/ACS, complementary solidarity health; FDEp, French Deprivation index; LPA, localized potential accessibility; GPs, general practitioners; LTD, long-term disease. ^a^All values were computed on the date of teleconsultation, and are expressed in terms of the teleconsultation unit: ie, “35.6% of teleconsultations were attended by men.”

 In total, 9.5% of TLC were addressed to beneficiaries of the complementary solidarity health. Furthermore, according to the deprivation index *Fdep*, the proportion of TLC decreased with each quintile, from the most to the least advantaged, with respectively 28.6% for Q1 and 14.7% for Q5. Regarding medical characteristics, 26.4% of teleconsultations attended by patients with LTD (Table 1).

 Geographical areas of residence revealed a predominant uptake among patients residing in large and intermediate urban areas (21.8%, 15.6%), whereas rural, widely scattered settlements beneficiaries constituted 8.9% of the population. In terms of GPs’ accessibility, 35.5% of TLCs were attended by beneficiaries living in a municipality listed in the two highest deciles and only 2.7% of TLC in the lowest decile. There was no correlation between the LPA indicator for GPs and the number of teleconsultations standardized on the population (*r* = -0.02; *P* =.8) (Table 1).

###  Prescribers’ Characteristics

 TLCs were predominantly performed by liberals (86.8%), followed by healthcare center-employed physicians (12.1%), and a minority by hospital-based practitioners. A slight decrease in the proportion of TLCs conducted by independent practitioners was observed during the study period, declining from 93.8% in the first semester of 2021 to 78.8% in the first semester of 2023. Conversely, healthcare center-employed physicians demonstrated a significant increase in TLC utilization, rising from 5.1% in the first semester of 2021 to 20.2% in the first semester of 2023 (Table 2).

**Table 2:** Characteristics of Prescribers Who Performed Teleconsultations

	**2021**		**2022**		**2023**	**Total**
	**S1**	**S2**	**S1**	**S2**	**S1**	
Teleconsultations^a^, N	2 648 520	1 917 246	2 517 230	2 061 691	2 057 678	11 202 364
Practice mode, N (%)						
Liberals	2 483 011 (93.8)	1 718 297 (89.6)	2 223 156 (88.3)	1 679 905 (81.5)	1 620 861 (78.8)	9 725 230 (86.8)
Health center-employed practitioners	133 950 (5.1)	178 283 (9.3)	268 634 (10.7)	361 623 (17.5)	415 292 (20.2)	1 357 782 (12.1)
Hospital practitioners	23 003 (0.9)	14 115 (0.7)	17 406 (0.7)	13 551 (0.7)	14 462 (0.7)	82 537 (0.7)
Missing data	8 556 (0.3)	6 551 (0.3)	8 034 (0.3)	6 612 (0.3)	7 063 (0.3)	36 816 (0.3)
Teleconsultation platform^b^, N (%)	112 407 (4.2)	199 709 (10.4)	312 296 (12.4)	493 122 (23.9)	580 315 (28.2)	1 697 849 (15.2)
Specialties of non- hospital practitioners, N (%)	2 616 961 (98.8)	1 896 580 (98.9)	2 491 790 (99.0)	2 041 528 (99.0)	2 036 153 (99.0)	11 083 012 (98.9)
General practitionner	2 147 258 (82.1)	1 456 253 (76.8)	1 906 007 (76.5)	1 428 216 (70.0)	1 360 080 (66.8)	8 297 814 (74.9)
Psychiatrist	168 868 (6.5)	137 493 (7.2)	167 999 (6.7)	138 567 (6.8)	142 689 (7.0)	755 616 (6.8)
Pediatrician	38 787 (1.5)	38 206 (2.0)	47 250 (1.9)	38 271 (1.9)	37 610 (1.8)	200 124 (1.8)
Dermatologist	31 928 (1.2)	20 612 (1.1)	24 368 (1.0)	17 963 (0.9)	20 233 (1.0)	115 104 (1.0)
Other specialties	208 618 (8.0)	226 854 (12.0)	324 867 (13.0)	399 933 (19.6)	456 364 (22.4)	1 616 636 (14.6)
Teleconsultation performed by the preferred doctor, N (%)						
Yes	1 316 691 (49.7)	832 209 (43.4)	1 162 115 (46.2)	791 968 (38.4)	746 748 (36.3)	4 849 731 (43.6)

^a^All values were computed on the date of teleconsultation, and are expressed in terms of the teleconsultation unit: ie, “86.8% of teleconsultations were conducted by liberals.” ^b^Teleconsultation platforms are used by both liberals and health centre-employed practitioners.

 We described a substantial increase in teleconsultations’ platform practice mode by health professionals, with utilization rates rising from 4.2% in the first semester of 2021 to 28.2% in the first semester of 2023 (Table 2).

 Regarding physician specialties, GPs carried out 74.9% of TLCs. The most frequently represented specialties were psychiatrists (6.8%), paediatricians (1.8%), and dermatologists (1.0%). Also, 43.6% of teleconsultations were performed by the patient preferred doctor (Table 2).

###  Dispensing Characteristics

 The 11 202 364 teleconsultations led to 39 223 498 dispensing over the study period. The average number of ATC-5 dispensed per teleconsultation was 2.53 (±1.95). Of all TLCs, 76.6% led to a single dispensing, 18.3% to 2 or 3 and 5.1% to more than 3 dispensing.

 Over half of TLCs (53.1%) resulted in class ATC-1 N drug dispensing, while 28.3% led to class ATC-1 A, 21.8% to class ATC-1 R and 21.1% to class ATC-1 J. Median time between TLC and first dispensing varied by drug class, most classes showed 0–1-day delays, while certain classes (ATC-1 B, C, G, and L) averaged were ≥3 days (Table 3).

**Table 3:** Number of Teleconsultations by ATC 1 Class Dispensed and Dispensing Characteristics

**ATC Level 1**		**Number of Teleconsultations With at Least** **One Dispensing**^a^**, N (%) **	**Time in Days**^b^**, Median [Q1, Q3]**
A	Alimentary tract and metabolism	3 174 502 (28.3)	1.0 [0.0; 9.4]
B	Blood and blood forming organs	792 906 (7.1)	3.2 [0.0; 29.4]
C	Cardiovascular system	1 158 166 (10.3)	4.0 [0.4; 34.8]
D	Dermatologicals	1 020 525 (9.1)	0.6 [0.0; 3.6]
G	Genito urinary system and sex hormones	781 011 (7.0)	2.8 [0.0; 33.8]
H	Systemic hormonal preparations	1 088 500 (9.7)	0.2 [0.0; 5.0]
J	Antiinfectives for systemic use	2 367 837 (21.1)	0.0 [0.0; 1]
L	Antineoplastic and immunomodulating agents	57 886 (0.5)	20.6 [2.0; 59.6]
M	Musculo-skeletal system	1 238 486 (11.1)	0.4 [0.0; 3.8]
N	Nervous system	5 953 236 (53.1)	0.6 [0.0; 5.0]
P	Antiparasitic products, insecticides and repellents	121 434 (1.1)	0.0 [0.0; 1.0]
R	Respiratory system	2 441 415 (21.8)	0.0 [0.0; 1.2]
S	Sensory organs	596 342 (5.3)	0.2 [0.0; 2.8]
	Others	508 569 (4.5)	NA

Abbreviations:ATC, Anatomical Therapeutic Chemical Classification; NA, not applicable. ^a^All values were computed on the date of teleconsultation, and are expressed in terms of the teleconsultation unit: ie, “28.3% of teleconsultations led to a dispensing of class A.” ^b^Time was calculated in days between the teleconsultation date and the first dispensing date.

 The proportion of TLCs by ATC-1 classes showed age-dependent variations. Patients under 18 years most frequently received class ATC-1 N (50.7%) and class ATC1-R (48.7%) medications. Patients ≥60 years primarily received class ATC-1 N (81.0%), class ATC-1 A (65.5%), and cardiovascular medications (58.0%) (Supplementary file 2, Table S2). Prescription patterns between liberal physicians and healthcare center employed physicians showed high similarity, except for cardiovascular medications (ATC-1 C), prescribed more frequently by liberals (Supplementary file 2, Table S3).

###  Classification of Teleconsultations According to the Context of Acute or Chronic Pathologies

 Based on the ATC-3 categorization on therapeutic indications (Table S1), TLCs were distributed as follows: 55.6% occurred in acute context, 42.8% in chronic context, and 1.6% remained unclassifiable. This distribution varied greatly according to age, with 80.3% of TLCs in an acute context among paediatric patients, compared with 55.0% among ≥60-year-olds. Most TLCs in the chronic context concerned the 18-59 and over-60 age groups. Regarding prescribers’ practice mode, respectively 53.2% and 75.3% of teleconsultations conducted by liberals and health centre-employed physicians were in acute context.

 The most ATC-3 classes dispensed in teleconsultation conducted in an acute context were analgesics (54.3%), respiratory drugs (18.5% of antihistamines for systemic use and 18.3% of decongestant), gastrointestinal medications (17.1% of vitamin A and D) and antibiotics (14.0% of beta-lactam and 7.3% of macrolides, lincosamides and streptogramins). In addition, almost one in ten teleconsultations conducted in an acute context resulted in the dispensing of analgesic opioids (9.0%). Most TLCs resulted in a single dispensing with a 0-1 day between teleconsultation and dispensing (Table 4).

**Table 4:** Number of Teleconsultations by the Top 10 Most Dispensed ATC33 Classes in Acute Context and Dispensing Characteristics

**ATC Level 3**	**No. of Teleconsultations** **With at Least One** **Dispensing**^a^**, N (%) **	**Time in Days**^b^, **Med [Q1, Q3]**	**Number of Dispensing Per Teleconsultation, N (%)**		
			**1**	**2**	**≥3**
Teleconsultations^a^, N	6 226 225 (55.6)				
N02B| Other analgesics and antipyretics	3 380 959 (54.3)	0.0 [0.0; 1.0]	3 028 971 (48.6)	226 329 (3.6)	125 658 (2.0)
R06A| Antihistamines for systemic use	1 151 132 (18.5)	0.8 [0.0; 8.8]	826 579 (13.3)	159 866 (2.6)	164 686 (2.6)
R01A| Decongestants and other nasal preparations for topical use	1 140 753 (18.3)	0.0 [0.0; 1.2]	1 045 362 (16.8)	58 357 (0.9)	3 7034 (0.6)
A11C| Vitamin A and D, including combinations of the two	1 063 753 (17.1)	1.8 [0.0; 21.0]	769 646 (12.4)	173 821 (2.8)	120 286 (1.9)
A02B|Drugs for peptic ulcer and gastro-oesophageal reflux disease	968 603 (15.6)	1.0 [0.0; 14.6]	627 722 (10.1)	138 413 (2.2)	202 467 (3.3)
M01A| Anti-inflammatory and antirheumatic products, non-steroids	940 542 (15.1)	0.2 [0.0; 2.4]	85 9424 (13.8)	52 563 (0.8)	28 555 (0.5)
J01C| Beta-lactam antibacterials, penicillins	872 609 (14.0)	0.0 [0.0; 0.6]	803 490 (12.9)	66 150 (1.1)	2 969 (0.0)
H02A| Corticosteroids for systemic use, plain	722 275 (11.5)	0.0 [0.0; 1.0]	690 547 (11.1)	19 349 (0.3)	12 379 (0.2)
N02A| Analgesic opioids	561 655 (9.0)	1.0 [0.0; 4.0]	402 576 (6.5)	91 568 (1.5)	67 511 (1.1)
A03A| Drugs for functional gastrointestinal disorders	549 121 (8.8)	0.0 [0.0; 1.6]	495 994 (8.0)	36 914 (0.6)	16 213 (0.3)

Abbreviation:ATC, Anatomical Therapeutic Chemical Classification. ^a^All values were computed on the date of teleconsultation, and are expressed in terms of the teleconsultation unit: ie, “15.6% of teleconsultations led to a dispensing of class A02B.” ^b^ Time was calculated in days between the teleconsultation date and the first dispensing date.

 Age-stratified analysis revealed distinct dispensing patterns between the paediatric and adult populations. In the paediatric population, the three most dispensed ATC-3 classes were “analgesics and antipyretics” (41.2%), “decongestants and other nasal preparations for topical use” (18.6%) and “antihistamines for systemic use” (15.8%). In the adult population, the three most dispensed ATC-3 classes were “analgesics and antipyretics” (29.7%), “antidepressants” (14.3%) and “anxiolytics” (11.3%) (Supplementary file 2, Table S4).

 TLC use in chronic context predominantly led to dispensing of antidepressants (28.1% of teleconsultations), anxiolytics (22.6%) and antipsychotics (6.6%). Moreover, 8.7% and 7.1% of TLCs led to dispensing “Lipid modifying agents, plain” and “Beta blocking agents” respectively. TLCs in the chronic context were characterised by a long median time between teleconsultation and dispensation (22 days for antithrombotic agents) and around a quarter of these resulted in 3 or more dispensations (Table 5, Supplementary file 3).

**Table 5:** Number of Teleconsultations by the Top 10 Most Dispensed ATC-3 Classes in Chronic Context and Dispensing Characteristics

**ATC Level 3**	**No. of Teleconsultations** **With at Least One** **Dispensing**^a^**, N (%) **	**Time in Days**^b^, **Med [Q1, Q3]**	**Number of Dispensing Per Teleconsultation, N (%)**		
			**1**	**2**	**≥3**
Teleconsultations^a^, N	**4 792 135 (42.8)**				
N06A| Antidepressants	1 346 161 (28.1)	8.0 [1.0; 36.0]	722 592 (15.1)	329 562 (6.9)	294 006 (6.1)
N05B| Anxiolytics	1 084 072 (22.6)	2.0 [0.0; 20.0]	777 441 (16.2)	178 662 (3.7)	127 968 (2.7)
R03A| Adrenergics, inhalants	556 702 (11.6)	1.0 [0.0; 9.8]	342 962 (7.2)	113 259 (2.4)	100 481 (2.1)
B01A| Antithrombotic agents	470 075 (9.8)	22.0 [1.0; 60.0]	172 740 (3.6)	114 720 (2.4)	182 615 (3.8)
C10B| Lipid modifying agents, plain	415 863 (8.7)	7.0 [1.0; 49.0]	241 079 (5.0)	93 333 (1.9)	81 451 (1.7)
G03A| Hormonal contraceptives for systemic use	381 680 (8.0)	36.5 [3.0; 92.0]	217 848 (4.5)	118 611 (2.5)	45 221 (0.9)
C07A| Beta blocking agents	338 767 (7.1)	11.0 [1.0; 57.0]	184 677 (3.9)	76 732 (1.6)	77 358 (1.6)
N05A| Antipsychotics	316 213 (6.6)	5.0 [1.0; 24.0]	185 796 (3.9)	78 613 (1.6)	51 803 (1.1)
A10B|Blood glucose lowering drugs, excluding insulins	298 335 (6.2)	8.6 [1.0; 44.4]	81 892 (1.7)	68 936 (1.4)	147 506 (3.1)
N03A| Antiepileptics	293 119 (6.1)	8.0 [1.0; 41.0]	146 613 (3.1)	68 579 (1.4)	77 927 (1.6)

Abbreviation:ATC, Anatomical Therapeutic Chemical Classification. ^a^All values were computed on the date of teleconsultation, and are expressed in terms of the teleconsultation unit: ie, “8.6% of teleconsultations led to a dispensing of class A02A.” ^b^ Time was calculated in days between the teleconsultation date and the first dispensing date.

 Over-60s had a higher rate of teleconsultations resulting in the dispensing of a “Cardiovascular system” drug (9.0% of TLC led to a dispensing of “Beta blocking agents” in the over-60s versus 1.7% in the 18-59 years), or “Blood and blood forming organs” (Supplementary file 2, Table S5).

## Discussion

###  Principal Results

 This study provided a comprehensive assessment of teleconsultation patterns and subsequent medication dispensing in France. We characterized the demographics of teleconsultation users, prescriber profiles, and dispensing patterns following teleconsultations for the 30 months following the COVID-19 pandemic.

 Half of teleconsultations led to the dispensing of drugs for acute indications (55.6%) and half to at least on dispensing of a drug for a chronic indication. Acute context was characterized by single medication dispensing, on the same day or on the following day, whereas chronic conditions distinguished by a longer delay between teleconsultation and first dispensing and multiple ATC-3 drug dispensing. Utilization patterns demonstrated age-related variations, but also variations according to practice mode of physicians. The majority of teleconsultation were attended by patients with good access to care and declared preferred doctor, contrary to the objective of this mode of care as described in the French Law (manage healthcare costs, combat medically underserved areas and facilitate local care for chronically ill patients).^29^

###  Patients’ Characteristics

 Teleconsultation was predominantly utilized by patients aged 18-59 years (69.3%), differing from traditional healthcare utilization patterns in France. Comparative analysis using OPEN DAMIR data (ie, public database, aggregated by the French health insurance, on reimbursements for all procedures by month)^30^ across the study period showed that this age group accounted for 49.7% of traditional consultations, while patients aged 60 and above represented 33.7% of face-to-face consultations compared to only 20.5% in our results. This underlines the fact that older people are less attracted on this type of consultation, probably due to the technological barriers involved.^31-33^ However, they were forced to use teleconsultation during COVID-19, explaining the decline observed in our results (from 25.9% in the first semester of 2021 to 17.7% in 2023).

###  Healthcare Accessibility

 Analysis of healthcare accessibility revealed that teleconsultations mostly occurred in major urban centres with high LPA. While this healthcare modality was implemented to address limited access to primary care in underserved areas,^4^ our findings showed no correlation between low LPA indicator. Three mains’ hypotheses may explain this pattern: first, limited access to in-person consultations due to physician unavailability within the patient’s desired timeframe or lack of registered preferred doctor in urban centers. Second, this care modality may be preferentially selected for convenience by residents and younger populations living mainly in urban centers. Third, healthcare providers in urban areas have rapidly adopted teleconsultations to manage patient volumes efficiently. Commercial platforms have facilitated this shift by offering user-friendly interfaces and streamlined processes. These factors, combined with the convenience and accessibility of telemedicine, have driven its increased use in urban settings.

###  Prescribers’ Characteristics

 Three medical specialties emerged: general practice, psychiatry, and pediatrics. This type of practice has become commonplace among GPs since COVID-19. In France, according to the study by Saint-Lary et al, 66.5% of GPs surveyed have increased their use of teleconsultation since this period.^9^ Teleconsultation also seems to be expanding in certain specialties, such as psychiatry. In the study by Johns et al, 51% of patients and 64% of clinicians surveyed considered teleconsultation to be very beneficial in improving access to regular psychiatric care,^34^ although there are still certain barriers for patients and professionals (reduced therapeutic alliance, difficulty in perceiving non-verbal communication).^35^ According to Open DAMIR data analysis, the three medical specialties conducting the highest number of consultations in France were general practice (71.1%), psychiatry (4.1%), and gynecology-obstetrics (2.9%). Our findings indicate increased teleconsultation utilization in pediatrics, predominantly for acute conditions (82.8%).

###  Emergence of Private Teleconsultation Platforms

 Finally, our study highlighted the emergence of private teleconsultation platform companies, increasing by 300% over our study period. Platform-based teleconsultations predominantly attended in acute context (79.0%) and resulted in dispensing of analgesics or anti-infectives. These companies only offer teleconsultations, enabling patients to book an appointment with a doctor they do not already know. In this way, since 2023, teleconsultation platforms have been required to present a certification,^26^ committing them to comply with health authorities’ recommendations, compliance assessment warrants future investigation.

###  Most Dispensed ATC-3 Drugs

 Analgesics constituted the most frequently dispensed medication category, with 30.2% of teleconsultations resulting in analgesic and antipyretic (N02B class) dispensing, predominantly in the pediatric population (41.9% of N02B dispensing). Notably, 5.0% of teleconsultations led to analgesic opioid (N02A class) dispensing, with 10.5% of these originating from platform-based teleconsultations, raising concerns regarding the appropriateness of remote prescribing for medications with significant misuse potential.

 Anti-infective dispensing represented 21.1% of teleconsultation prescriptions, despite certain cases requiring diagnostic testing (bacterial confirmation) or strain-specific sensitivity testing to guide appropriate therapy. In the context of increasing antimicrobial resistance, the prescription of antibiotics without face-to-face consultations should be studied in terms of compliance with good dispensing practices. One can postulate that the prescription of an antibiotic is adversely influenced by the lack of possibility for a clinical examination in acute infectious situations, such as cough. Certain ATC-3 classes, including cardiovascular and nervous system medications, were typically associated with chronic context. While teleconsultation may facilitate medication renewals for ongoing patient management, the appropriateness of treatment initiation via teleconsultation requires careful consideration.

###  Strengths and Limitations

 The national scale of the cohort represents a key strength of this study, enabling more comprehensive assessment of teleconsultation utilization through the characterization of patients, prescribers and dispensing. However, this study presents several limitations. The technical inability to analyze the complete national consultation dataset due to its volume limited our comparative analysis capabilities between outcomes in teleconsultation and face-to-face consultation. We then have chosen to use aggregated data from French national health insurance as a comparator. With regard to the medical characteristics of patients, relying on long-term illness status to identify chronic diseases underestimates the use of teleconsultation by patients with chronic diseases in our study population. Regarding post-teleconsultation medication dispensing analysis, the absence of prescription data in the *SNDS* likely underestimates the actual number of prescriptions issued. This underestimation presumably affects predominantly acute care teleconsultation assessments. Due to the absence of consultation reasons and medication indications in the *SNDS*, we have relied onproxy indicators based on ATC medication classes to classified teleconsultations context as ‘chronic condition context’ versus “acute condition context.” This approach is debatable since medications are often used out of proper Marketing Authorization documentation and by classifying teleconsultations as chronic whenever they included at least one chronic indication, we may have underestimated the extent of TLC use for acute reasons. However, results of median time intervals between teleconsultation and initial dispensing, along with the number of dispensing per teleconsultation were consistent with our classification.

###  Implications for Health Policy

 First, our results suggest that TLC are missing their intended target in addressing healthcare access disparities. The profile of TLC users, predominantly younger, urban, and well-connected individuals, highlight the need for targeted interventions. Financial incentives for healthcare professionals in underserved areas (low LPA) could help redirect this resource toward target populations (older adults or rural communities, who currently remain underrepresented). Second, to ensure continuity and coordination of care, TLC should be prioritized for chronic disease follow-up, with a formal requirement to link consultations to a patient’s declared preferred doctor. TLC might be restricted to cases where an initial in-person consultation has already taken place. Third, the rapid expansion of private TLC platforms necessitates stricter oversight. Mandatory certification, aligned with the Haute Autorité de Santé framework and the guidelines of the Ordre National des Médecins, is essential to guarantee quality and safety. Additionally, limiting the use of these platforms to non-urgent care could mitigate risks of care fragmentation and overuse. Fourth, the study underscores the urgency of regulating prescription practices, particularly for antibiotics and opioid analgesics. National guidelines should be developed and enforced to ensure appropriate prescribing, reducing the potential for misuse and antimicrobial resistance. This work calls for the establishment of a comprehensive primary care health data repository in France, to allow deeper analysis of teleconsultation usage (including reasons, outcomes, and regional disparities) while facilitating ongoing evaluation of care quality, cost-effectiveness, and equity in access.

## Conclusion

 This study underlined that teleconsultation is predominantly utilized by young, urban patients with good access to healthcare, contrary to the objectives outlined in French legislation. The dispensing of antibiotics or other medications with a substantial risk of misuse, sometimes through commercial platforms, raises concerns regarding prescription appropriateness. These findings highlight the need for further investigation to inform regulatory frameworks and establish clearer clinical guidelines for teleconsultation use, thereby ensuring its effective and safe contribution to patient care.

## Disclosure of artificial intelligence (AI) use

 Not applicable.

## Ethical issues

 Not applicable (French nationwide health insurance databases are used within the framework of regulatory agreements).

## Conflicts of interest

 Authors declare that they have no conflicts of interest.

## Disclaimer

 The views expressed are those of the authors and do not necessarily reflect the position of the relevant health authorities.

## Supplementary files


Supplementary file 1 contains RECORD-PE checklist.

Supplementary file 2 contains Tables S1-S5.

Supplementary file 3 contains the visual abstract.
