# Supplementary material for: Medication Dispensing Patterns Following Teleconsultations in France From January 2021 to June 2023: A Retrospective Cohort Study Using the National Health Data System
Source: Int J Health Policy Manag. 2026 May 24;15:9215. doi: 10.34172/ijhpm.9215 (PMC13498963; doi:10.34172/ijhpm.9215)
Supplement: Supplementary file 2 [file ijhpm-15-9215-s002.pdf]

Article title: Medication Dispensing Patterns Following Teleconsultations in France From January 2021 to June 2023: A Retrospective Cohort Study Using the National Health Data System

Journal name: International Journal of Health Policy and Management (IJHPM)

Authors' information: Julie Rigoureau[1]*, François Bettega[1], Marc Chanelière[1,2], Teddy Novais[1,3], Stéphane Sanchez[4,5], Stéphanie Sidorkiewicz[6,7], Aurélie Moskal[1,8], Marie Viprey[1,8]

[1]Research on Healthcare Performance (RESHAPE), INSERM U1290, Université Claude Bernard Lyon 1, Lyon, France.

[2]Collège universitaire de Médecine Générale, Université Lyon 1, Villeurbanne, France.

[3]Service Pharmacie, Hôpital des Charpennes, Hospices Civils de Lyon, Villeurbanne, France.

[4]Pôle Territorial Santé Publique, Hôpitaux Champagne Sud, Troyes, France.

[5]Faculté de Médecine, Université de Reims Champagne Ardennes, Reims, France.

[6]Centre for Research in Epidemiology and Statistics (CRESS), Université Paris Cite and Université Sorbonne Paris Nord, Inserm, INRAE, Paris, France.

[7]Department of General Practice, Université Paris Cité, Paris, France.

[8]Hospices Civils de Lyon, Service des Données de Santé, Lyon, France.

***Correspondence to:** Julie Rigoureau; Email: julie.rigoureau@inserm.fr

Citation: Rigoureau J, Bettega F, Chanelière M, et al. Medication dispensing patterns following teleconsultations in France from January 2021 to June 2023: a retrospective cohort study using the national health data system. Int J Health Policy Manag. 2026;15:9215. doi:10.34172/ijhpm.9215

Supplementary file 2

Tables S1-S5

Table S1: Classification of ATC-3 classes according to treatment indications by acute or chronic disease context

ATC 3 – Acute disease context	ATC 3 – Chronic disease context
**« Alimentary tract and metabolism» class**	
All ATC-3 in the "Alimentary tract and metabolism" class except: A05A, A07E, A10A and A10B	A05A: Bile therapy<br>A07E: Intestinal anti-inflammatory agents<br>A10A: Insulins and analogues<br>A10B: Blood glucose lowering drugs, excl. insulins
**« Blood and blood forming organs»**	
B02A: Antifibrinolytics<br>B03A: Iron preparations<br>B03B: Vitamin b12 and folic acid	B01A: Antithrombotic agents<br>B05B: I.V. solutions
**« Cardiovascular system » class**	
	All ATC-3
**« Dermatologicals » class**	

D01A: Antifungals for topical use<br>D01B: Antifungals for systemic use<br>D02A: Emollients and protectives<br>D05A: Antipsoriatics for topical USE<br>D06A: Antibiotics for topical use<br>D07A: Corticosteroids, plain<br>D07X: Corticosteroids, other combinations<br>D08A: Antiseptics and disinfectants	D06B: Chemotherapeutics for topical use<br>D10A: Anti-acne preparations for topical use<br>D10B: Anti-acne preparations for systemic use
**« Genito urinary system and sex hormones » class**	
G01A: Antiinfectives and antiseptics, excl. combinations with corticosteroids<br>G04B: Urologicals	G02B: Contraceptives for topical use<br>G03A: Hormonal contraceptives for systemic use<br>G03B: Androgens<br>G03C: Estrogens<br>G03D: Progestogens<br>G03E: Androgens and female sex hormones in combination<br>G03G: Gonadotropins and other ovulation stimulants<br>G04C: Drugs used in benign prostatic hypertrophy
**« Systemic hormonal preparations, excl. sex hormones and insulins » class**	
H02A: Corticosteroids for systemic use, plain	H01A: Anterior pituitary lobe hormones and analogues<br>H03A: Thyroid preparations<br>H03B: Antithyroid preparations
**« Antiinfectives for systemic use » class**	
All ATC-3 in the “Antiinfectives for systemic use” class except J05A	J05A: Direct acting antivirals
**« Musculo-skeletal system » Class**	
M01A: Antiinflammatory and antirheumatic products, non-steroids<br>M02A: Topical products for joint and muscular pain	M03B: Muscle relaxants, centrally acting agents<br>M04A: Antigout preparations<br>M05B: Drugs affecting bone structure and mineralization
**« Nervous system » class**	
N02A: Opioids<br>N02B: Other analgesics and antipyretics<br>N05C: Hypnotics and sedatives	N02C: Antimigraine preparations<br>N03A: Antiepileptics<br>N04A: Anticholinergic agents<br>N04B: Dopaminergic agents

	N05A: Antipsychotics<br>N05B: Anxiolytics<br>N06A: Antidepressants<br>N06B: Psychostimulants, agents used for adhd and nootropics<br>N07B: Drugs used in addictive disorders
**« Respiratory system » class**	
R01A: Decongestants and other nasal preparations for topical use<br>R02A: Throat preparations<br>R05C: Expectorants, excl. combinations with cough suppressants<br>R05D: Cough suppressants, excl. combinations with expectorants<br>R06A: Antihistamines for systemic use<br>R07A: Other respiratory system products	R03A: Adrenergics, inhalants<br>R03B: Other drugs for obstructive airway diseases, inhalants<br>R03D: Other systemic drugs for obstructive airway diseases
**« Sensory organs » class**	
All ATC-3 in the "sensory organs" class except S01E	S01E: Antiglaucoma preparations and miotics

Table S2: Number of teleconsultations by ATC-1 class by age group

		Number of teleconsultations with at least one dispensing [a], N (%)		
		< 18 years	*18 -59 years*	*≥60 years*
**Teleconsultations, N**		**1 129 981**	**7 769 124**	**2 303 260**
**ATC-1 class, N (%)**				
**A**	**Alimentary tract and metabolism**	370 080 (32.8)	2 950 785 (38.0)	1 508 595 (65.5)
**B**	**Blood and blood forming organs**	23 012 (2.0)	406 836 (5.2)	458 389 (19.9)
**C**	**Cardiovascular system**	5 703 (0.5)	688 158 (8.9)	1 336 161 (58.0)
**D**	**Dermatologicals**	194 147 (17.2)	864 334 (11.1)	222 072 (9.6)
**G**	**Genito urinary system and sex hormones**	14 844 (1.3)	705 836 (9.1)	153 284 (6.7)
**H**	**Systemic hormonal preparations**	112 981 (10.0)	737 569 (9.5)	259 835 (11.3)
**J**	**Antiinfectives for systemic use**	317 779 (28.1)	1 689 747 (21.7)	457 810 (19.9)

**L**	**Antineoplastic and immunomodulating agents**	603 (0.1)	34 889 (0.4)	27 569 (1.2)
**M**	**Musculo-skeletal system**	64 540 (5.7)	1 038 038 (13.4)	306 224 (13.3)
**N**	**Nervous system**	572 772 (50.7)	5 988 381 (77.1)	1 864 640 (81.0)
**P**	**Antiparasitic products, insecticides and repellents**	21 631 (1.9)	112 952 (1.5)	16 617 (0.7)
**R**	**Respiratory system**	550 026 (48.7)	2 504 838 (32.2)	501 397 (21.8)
**S**	**Sensory organs**	175 704 (15.5)	434 687 (5.6)	117 762 (5.1)
	**Others**	69 528 (6.2)	294 535 (3.8)	62 344 (2.7)

[a]The percentage of teleconsultations by ATC 1 class was calculated by age group, i.e. on the number of teleconsultations by age group

Table S3: Number of teleconsultations by ATC-1 class by practice mode group

		**Number of teleconsultations with at least one dispensing [a], N (%)**			
		Liberals	*Hospital practitionners*	*Health-centre employed*	*Teleconsultation Platforms [b]*
**Teleconsultations, N**		**9 725 230**	**82 537**	**1 357 782**	**1 697 849**
**ATC-1 class, N (%)**					
**A**	**Alimentary tract and metabolism**	2 824 235 (29.0)	25 053 (30.4)	319 504 (23.5)	379 222 (22.3)
**B**	**Blood and blood forming organs**	741 700 (7.6)	14 007 (17.0)	35 750 (2.6)	103 646 (6.1)
**C**	**Cardiovascular system**	1 106 348 (11.4)	6 053 (7.3)	44 444 (3.3)	45 083 (2.7)
**D**	**Dermatologicals**	884 807 (9.1)	3 630 (4.4)	128 333 (9.5)	178 386 (10.5)

**G**	**Genito urinary system and sex hormones**	680 516 (7.0)	10 594 (12.8)	86 875 (6.4)	87 211 (5.1)
**H**	**Systemic hormonal preparations**	947 368 (9.7)	11 526 (14.0)	126 649 (9.3)	176 189 (10.4)
**J**	**Antiinfectives for systemic use**	1 947 093 (20.0)	7 434 (9.0)	410 863 (30.3)	568 491 (33.5)
**L**	**Antineoplastic and immunomodulating agents**	41 969 (0.4)	14 084 (17.1)	1 049 (0.1)	774 (0.0)
**M**	**Musculo-skeletal system**	1 073 395 (11.0)	6 007 (7.3)	157 711 (11.6)	215 734 (12.7)
**N**	**Nervous system**	5 172 896 (53.2)	31 218 (37.8)	727 170 (53.6)	936 147 (55.1)
**P**	**Antiparasitic products, insecticides and repellents**	100 643 (1.0)	483 (0.6)	19 950 (1.5)	20 635 (1.2)
**R**	**Respiratory system**	2 070 589 (21.3)	3 654 (4.4)	363 539 (26.8)	475 319 (28.0)
**S**	**Sensory organs**	492 298 (5.1)	747 (0.9)	102 111 (7.5)	148 471 (8.7)
	**Others**	423 388 (4.4)	4 895 (5.9)	79 616 (5.9)	103 646 (6.1)

[a]The percentage of teleconsultations by ATC 1 class was calculated by practice mode group, i.e., on the number of teleconsultations by practice mode group

[b] The teleconsultation platform mode is independent of professional practice mode

Table S4: Number of teleconsultations by ATC-3 class by age group

		**Number of teleconsultations with at least one dispensing [a], N (%)**		
		< 18 years	*18 -59 years*	*≥60 years*
**Teleconsultations, N**		**1 129 981**	**7 769 124**	**2 303 260**
**ATC-3 class, N (%)**				
**A| Alimentary tract and metabolism**				
A02B	Drugs for peptic ulcer and gastro-oesophageal reflux disease	14 406 (1.3)	604 367 (7.8)	349 829 (15.2)
A03A	Drugs for functional gastrointestinal disorders	66 873 (5.9)	407 430 (5.2)	74 818 (3.2)
A10B	Blood glucose lowering drugs, excl. insulins	79 (0.0)	114 029 (1.5)	184 226 (8.0)
A11C	Vitamin A and D, incl. Combinations of the two	87 833 (7.8)	644 397 (8.3)	331 523 (14.4)
**B| Blood and blood forming organs**				

B01A	Antithrombotic agents	567 (0.1)	129 749 (1.7)	339 759 (14.8)
**C| Cardiovascular system**				
C07A	Beta blocking agents	528 (0.0)	131 205 (1.7)	207 034 (9.0)
C10B	Lipid modifying agents, plain	32 (0.0)	62 684 (0.8)	136 290 (5.9)
**D| Dermatologicals**				
D01A	Antifungals for topical use	26 942 (2.4)	238 206 (3.1)	51 779 (2.2)
D07A	Corticosteroids, plain	41 574 (3.7)	191 193 (2.5)	52 535 (2.3)
**G| Genito urinary system and sex hormones**				
G03A	Hormonal contraceptives for systemic use	11 364 (1.0)	370 259 (4.8)	57 (0.0)
**H| Systemic hormonal preparations**				
H02A	Corticosteroids for systemic use, plain	108 403 (9.6)	497 039 (6.4)	116 833 (5.1)
H03A	Thyroid preparations	1 443 (0.1)	205 778 (2.6)	133 265 (5.8)
**J| Antiinfectives for systemic use**				
J01C	Beta-lactam antibacterials, penicillins	151 487 (13.4)	573 687 (7.4)	147 435 (6.4)
J01F	Macrolides, lincosamides and streptogramins	32 382 (2.9)	342 915 (4.4)	80 378 (3.5)
J01X	Other antibacterials	5 860 (0.5)	271 886 (3.5)	72 445 (3.1)
**M| Musculo-skeletal system**				
M01A	Antiinflammatory and antirheumatic products, non-steroids	57 609 (5.1)	756 496 (9.7)	126 437 (5.5)
M02A	Topical products for joint and muscular pain	6 351 (0.6)	210 939 (2.7)	88 884 (3.9)
**N| Nervous system**				
N02A	Opioids	3 303 (0.3)	408 103 (5.3)	170 220 (7.4)
N02B	Other analgesics and antipyretics	465 984 (41.2)	2 394 226 (30.8)	597 357 (25.9)
N03A	Antiepileptics	5 038 (0.4)	217 722 (2.8)	96 861 (4.2)
N05A	Antipsychotics	8 511 (0.8)	289 648 (3.7)	79 639 (3.5)
N05B	Anxiolytics	9 003 (0.8)	850 467 (10.9)	285 561 (12.4)
N05C	Hypnotics and sedatives	8 086 (0.7)	332 288 (4.3)	207 192 (9.0)
N06A	Antidepressants	10 282 (0.9)	1 119 007 (14.4)	323 625 (14.1)
**R| Respiratory system**				
R01A	Decongestants and other nasal preparations for topical use	210 702 (18.6)	816 714 (10.5)	113 337 (4.9)
R03A	Adrenergics, inhalants	74 400 (6.6)	371 890 (4.8)	110 412 (4.8)

R05D	Cough suppressants, excl. Combinations with expectorants	22 962 (2.0)	228 310 (2.9)	42 772 (1.9)
R06A	Antihistamines for systemic use	179 059 (15.8)	822 513 (10.6)	149 559 (6.5)

[a]The percentage of teleconsultations by ATC 3 class was calculated by age group, i.e, on the number of teleconsultations by age group

Table S5: Number of teleconsultations by ATC-3 class by practice mode group

		Number of teleconsultations with at least one dispensing [a], N (%)			
		Liberals	*Hospital practitionners*	*Health-centre employed*	*Teleconsultation Platforms* [b]
**Teleconsultations, N**		**9 725 230**	**82 537**	**1 357 782**	**1 697 849**
**ATC-3 class, N (%)**					
**A| Alimentary tract and metabolism**					
A02B	Drugs for peptic ulcer and gastro-oesophageal reflux disease	890 080 (9.2)	6 290 (7.6)	70 841 (5.2)	81 053 (4.8)
A03A	Drugs for functional gastrointestinal disorders	443 352 (4.6)	2 173 (2.6)	103 126 (7.6)	132 090 (7.8)
A10B	Blood glucose lowering drugs, excl. insulins	283 932 (2.9)	1 684 (2.0)	12 119 (0.9)	11 036 (0.6)
A11C	Vitamin A and D, incl. Combinations of the two	991 545 (10.2)	8 597 (10.4)	61 857 (4.6)	52 754 (3.1)

**B| Blood and blood forming organs**					
B01A	Antithrombotic agents	450 212 (4.6)	4 573 (5.5)	14 796 (1.1)	12 787 (0.8)
**C| Cardiovascular system**					
C07A	Beta blocking agents	323 712 (3.3)	1 743 (2.1)	12 960 (1.0)	12 700 (0.7)
C10B	Lipid modifying agents, plain	399 404 (4.1)	2 093 (2.5)	13 991 (1.0)	12 717 (0.7)
**D| Dermatologicals**					
D01A	Antifungals for topical use	272 139 (2.8)	464 (0.6)	43 520 (3.2)	46 292 (2.7)
D07A	Corticosteroids, plain	253 638 (2.6)	784 (0.9)	30 048 (2.2)	36 128 (2.1)
**G| Genito urinary system and sex hormones**					
G03A	Hormonal contraceptives for systemic use	331 479 (3.4)	993 (1.2)	47 945 (3.5)	48 935 (2.9)
**H| Systemic hormonal preparations**					
H02A	Corticosteroids for systemic use, plain	60 1848 (6.2)	4 865 (5.9)	114 404 (8.4)	165 852 (9.8)
H03A	Thyroid preparations	324 761 (3.3)	2 751 (3.3)	11 628 (0.9)	10 057 (0.6)
**J| Antiinfectives for systemic use**					
J01C	Beta-lactam antibacterials, penicillins	687 932 (7.1)	1 228 (1.5)	182 844 (13.5)	271 998 (16.0)
J01F	Macrolides, lincosamides and streptogramins	385 750 (4.0)	587 (0.7)	69 023 (5.1)	95 067 (5.6)
J01X	Other antibacterials	277 978 (2.9)	338 (0.4)	71 701 (5.3)	89 376 (5.3)
**M| Musculo-skeletal system**					
M01A	Antiinflammatory and antirheumatic products, non-steroids	798 316 (8.2)	4 079 (4.9)	137 131 (10.1)	192 774 (11.4)
M02A	Topical products for joint and muscular pain	270 150 (2.8)	217 (0.3)	35 731 (2.6)	44 721 (2.6)
**N| Nervous system**					
N02A	Opioids	508 523 (5.2)	5 038 (6.1)	47 607 (3.5)	58 712 (3.5)
N02B	Other analgesics and antipyretics	2 789 778 (28.7)	9 444 (11.4)	579 067 (42.6)	779 249 (45.9)
N03A	Antiepileptics	276 451 (2.8)	5 295 (6.4)	8 188 (0.6)	7 527 (0.4)
N05A	Antipsychotics	297 252 (3.1)	2 105 (2.6)	11 314 (0.8)	11 035 (0.6)
N05B	Anxiolytics	1 009 450 (10.4)	4 620 (5.6)	62 012 (4.6)	64 529 (3.8)
N05C	Hypnotics and sedatives	511 896 (5.3)	2 017 (2.4)	22 023 (1.6)	20 171 (1.2)
N06A	Antidepressants	1 266 001 (13.0)	6 219 (7.5)	59 706 (4.4)	56 988 (3.4)
**R| Respiratory system**					
R01A	Decongestants and other nasal preparations for topical use	922 501 (9.5)	560 (0.7)	216 727 (16.0)	286 785 (16.9)
R03A	Adrenergics, inhalants	496 453 (5.1)	1 286 (1.6)	58 179 (4.3)	72 273 (4.3)

R05D	Cough suppressants, excl. Combinations with expectorants	253 555 (2.6)	20 (0.0)	40 431 (3.0)	55 068 (3.2)
R06A	Antihistamines for systemic use	1 009 430 (10.4)	2 293 (2.8)	137 012(10.1)	171 950 (10.1)

[a]The percentage of teleconsultations by ATC 1 class was calculated by practice mode group, i.e., on the number of teleconsultations by practice mode group

[b] The teleconsultation platform mode is independent of professional practice mode
