# Supplementary material for: Medication Dispensing Patterns Following Teleconsultations in France From January 2021 to June 2023: A Retrospective Cohort Study Using the National Health Data System
Source: Int J Health Policy Manag. 2026 May 24;15:9215. doi: 10.34172/ijhpm.9215 (PMC13498963; doi:10.34172/ijhpm.9215)
Supplement: Supplementary file 3 [file ijhpm-15-9215-s003.pdf]

Article title: Medication Dispensing Patterns Following Teleconsultations in France From January 2021 to June 2023: A Retrospective Cohort Study Using the National Health Data System

Journal name: International Journal of Health Policy and Management (IJHPM)

Authors' information: Julie Rigoureau[1]*, François Bettega[1], Marc Chanelière[1,2], Teddy Novais[1,3], Stéphane Sanchez[4,5], Stéphanie Sidorkiewicz[6,7], Aurélie Moskal[1,8], Marie Viprey[1,8]

[1]Research on Healthcare Performance (RESHAPE), INSERM U1290, Université Claude Bernard Lyon 1, Lyon, France.

[2]Collège universitaire de Médecine Générale, Université Lyon 1, Villeurbanne, France.

[3]Service Pharmacie, Hôpital des Charpennes, Hospices Civils de Lyon, Villeurbanne, France.

[4]Pôle Territorial Santé Publique, Hôpitaux Champagne Sud, Troyes, France.

[5]Faculté de Médecine, Université de Reims Champagne Ardennes, Reims, France.

[6]Centre for Research in Epidemiology and Statistics (CRESS), Université Paris Cite and Université Sorbonne Paris Nord, Inserm, INRAE, Paris, France.

[7]Department of General Practice, Université Paris Cité, Paris, France.

[8]Hospices Civils de Lyon, Service des Données de Santé, Lyon, France.

***Correspondence to:** Julie Rigoureau; Email: julie.rigoureau@inserm.fr

Citation: Rigoureau J, Bettega F, Chanelière M, et al. Medication dispensing patterns following teleconsultations in France from January 2021 to June 2023: a retrospective cohort study using the national health data system. Int J Health Policy Manag. 2026;15:9215. doi:10.34172/ijhpm.9215

Supplementary file 3. Visual Abstract

MEDICATION DISPENSING PATTERNS FOLLOWING TELECONSULTATIONS IN FRANCE

Context and Method

CONTEXT

Teleconsultation (TLC) grew significantly

Medication dispensing patterns following TLC
→ largely unexplored

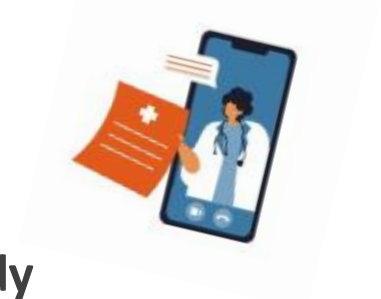

METHOD

Retrospective cohort study

French health database *(SNDS)*

TLCs **executed by physician**
01/01/2021 – 06/30/2023
Resulting in at least **one dispensing within 6 months**

Patients and prescribers

N = 11,202,364 TLCs

PATIENTS' CHARACTERISTICS

Young, urban and good access to care

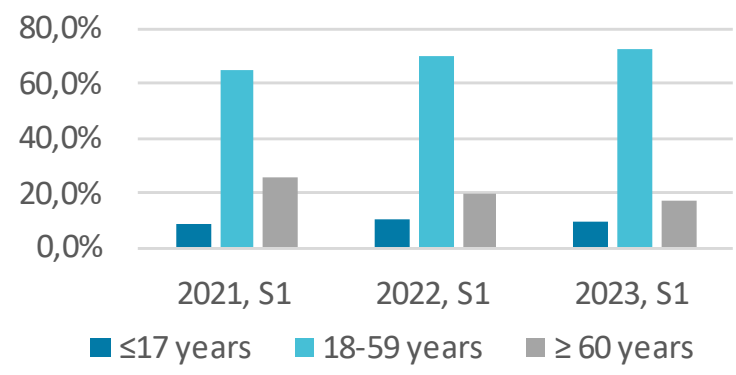

PRESCRIBERS' CHARACTERISTICS

Practice mode
Liberals: 86.8% of TLCs

Teleconsultation platform

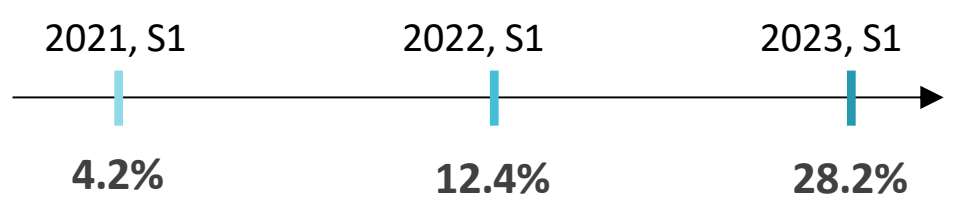

Dispensing

TWO CONTEXTS OF DISPENSING

Chronic indication, n =4,792,135 (42.8% of TLCs)

ATC level 3	Teleconsultations with at least one dispensing, N (%)
N06A| Antidepressants	1 346 161 (28.1)
N05B| Anxiolytics	1 084 072 (22.6)
R03A| Adrenergics, inhalants	556 702 (11.6)

Acute indication, n = 6,226,225 (55,6% TLCs)

ATC level 3	Teleconsultations with at least one dispensing, N (%)
N02B| Other analgesics and antipyretics	3 380 959 (54.3)
R06A| Antihistamines for systemic use	1 151 132 (18.5)
R01A| Decongestants and other nasal preparations	1 140 753 (18.3)

IMPULSE Epidémiologie des produits de santé · Inserm · Lyon 1 · HCL · Reshape Research on healthcare performance
